# Supplementary material for: Are carbon emissions trading and green financial instruments synergistic? -Comprehensive quantitative research based on content analysis
Source: PLoS One. 2024 Mar 7;19(3):e0298601. doi: 10.1371/journal.pone.0298601 (PMC10919601; doi:10.1371/journal.pone.0298601)
Supplement: S1 Data — (ZIP) [file pone.0298601.s001.zip › Data/地级市Z_F工作报告.zip!/╣π╢1⁄2/╔╪╣╪╩╨/└┤╫╘╣1⁄2╓┌║┼í╛┬φ┐╦╩2╛▌═°í┐_2015.docx]

各位代表：

　　我代表市人民政府向大会作工作报告，请予审议，并请政协各位委员和其他列席 人员提出意见。

2014年工作回顾

　　2014年，在省委、省政府和市委的正确领导下，在市人大及其常委会和市政协的 监督支持下，市政府突出“三大抓手”，落实莞韶对口帮扶工作要求，着力加快我市 绿色转型振兴发展，全力完成市十三届人大四次会议确定的任务。

　　经济发展稳中有进。据统计，全市生产总值1111. 5亿元，增长9. 5%，增幅高于 全省平均水平。物价涨幅低于全省平均水平。城镇、农村居民人均可支配收入分别增 长9. 3%、10.2%，城乡居民收入差距继续缩小。全面建成小康社会实现程度80%， 比2013年提高1.4个百分点。全市地方公共财政预算收入81. 98亿元，增长14. 2%。 内需扩大，金融外贸稳定增长。东莞银行、东北证券、前海人寿进驻韶关，首家民营 企业在香港上市。固定资产投资完成746. 7亿元，增长18.3%。农业增产增效，工业 平稳发展，服务业提升发展。国家重点农业龙头企业现“零”的突破。新增国家 4A级景区2个，旅游总收入增长20. 3%。三次产业结构优化为12. 9： 40.6： 46.5。 民营经济和县域经济占比分别提高1个、2个百分点。“三大抓手”成效明显，省级 产业园规模以上工业增加值、税收收入占比分别提高3.1个、1.4个百分点。东莞与 韶关对口帮扶工作成效显著，“一园一城七组团”合计签约项目15个，总投资 316. 9亿元，其中开工107个，投资101. 7亿元；莞韶城一期初具规模；莞韶两市 “十大”领域合作进展顺利。新增国家高新技术企业6家、省级工程技术研发中心2 家，专利申请量、授权量连续10年居全省山区市首位，创新驱动更加有力。

　　城乡建设协调推进。交通基础设施建设力度加大。乐广高速公路和赣韶铁路建成 运营，大广高速公路建设加快，新港码头港澳航线复航。全长88公里的环城高速公 路建成贯通，我市成为粤东西北地区首个、全省第四个有环城高速公路的城市。新增 铁路17公里、高速公路162公里，改造国省道50公里，建成通自然村公路530公 里。中心城市扩容提质加快推进。市区新建改建市政道路1条。芙蓉新城路网全面 动工，安置新村建设全面铺开；市民文化活动中心、综合客运枢纽扎实推进；人保财 险等企业进驻新城总部经济区，新城碧桂园酒店建成。在老城区，工业西片区内涝整 治工程基本完工；东环商贸城建设加快，百年东街建成开业，“三旧”改造有序开展。 县城和中心镇集聚人口和产业能力增强。拆除违法建筑52万平方米，城乡建设更加 有序。我市跻身国家生态文明先行示范区行列，国家节能减排财政综合政策示范城市 建设有序推进，生态建设和节能减排成效明显。完成造林41. 26万亩，森林碳汇造林 24. 76万亩。韶钢二氧化硫减排工程竣工；淘汰黄标车及老旧车10465辆；市区更新 投放LNG新能源公交车100辆、纯电动出租车50辆、CNG出租车380辆。芙蓉新城 污水处理厂建成投产，田螺冲污水处理厂扎实推进；完成畜禽养殖场减排工程377 个。完成市区LED路灯改造。单位GDP能耗下降4. 76%。完成节能减排和大气污染 防治年度目标任务。

　　民生保障持续加强。民生支出占财政总支出的比重提高1.2个百分点，基本公共 服务均等化水平继续提高，社会保障体系不断完善。城镇登记失业率2. 42%，农村劳 动力技能培训转移就业工作连续六年进入全省优秀行列。人均养老金、城乡居保基础 养老金、底线民生保障标准增幅明显，城乡医保一体化建设扎实推进。完成农村低收 入困难户住房改造3687户。开工建设保障性住房7596套、建成7495套，原曲仁矿 棚改基本完成，在主城区东部初步形成一个可容纳数万人居住、配套设施较完善的城 市新组团。韶关实验中学和实验小学开学，市一中运动场工程竣工，北江中学综合楼 加快建设。我市成为粤东西北地区首个省教育强县（市、区）10%覆盖的省教育强 市。市中医院晋级三甲中医院，县级公立医院全部取消药品加成，人均基本公共卫生 服务经费大幅提高。实施“单独二孩”政策。完成国家和省文化惠民工程任务，广泛 开展“北江源•韶韵风”和“文明和谐•舞动韶城”系列群众文化活动，完成市直 文化院团改革和《南叶》杂志转企改制工作，广播电视台全面深化改革初见成效，新 兴媒体等文化阵地建设进一步拓展。培育地方文化品牌有新成效，乳源瑶族自治县被 命名为中国民间文化艺术之乡，瑶族民歌入选国家级非物质文化遗产代表性项目扩展 名录，小说《水滴》和歌曲《恩情》荣获省“五个一工程”奖，《民生关注》栏目 荣获2014年中国城市电视台品牌20强。全国文明城市创建有序推进。建成20个乡 镇农民体育健身工程，免费向社会开放25万平方米机关事业单位体育场馆。节俭举 办市第十四届运动会，在省第五届民运会上获团体总分第三名。乳源瑶族自治县被评 为全国民族团结进步模范集体。顺利完成村（居）“两委”换届选举工作。安全生产 完成省下达的控制目标。食品药品安全、“平安韶关”创建和“两建”等工作取得新 进展，社会和谐稳定。在省综治创平考核中名列第一，群众安全感测评位居全省第 四。新一轮扶贫开发“双到”工作成效明显。市主城区建成小公园、小广场、小运动 场、小停车场、小菜市场共1个，市政府公开承诺办好的8件民生实事和市政府所 属单位承诺的51件实事基本完成。

　　政府建设明显加强。提高依法行政水平，深入推进法治政府建设。认真办理人大 代表建议、政协提案。全面完成市、县级政府机构改革。初步构建实体大厅、网上办 事大厅和掌上政务大厅“三位一体”的政务服务体系。2014年市级保留行政审批项 目比2011年底压减了 48. 92%。编制政府部门权责清单试点工作稳步推进。工商登记 制度改革成效显著，全市登记各类市场主体12. 4万户，注册资本875. 8亿元，分别 比上年增加10365户和164亿元。全面实施建设工程交易网上报名和资格后审制度。 市重点项目投资建设直接落地改革试点、公共资源交易制度改革、投融资体制改革和 以南雄市为试点的行政执法体系改革扎实推进。建立全口径预算编报体系。组建市国 投资产开发公司，优化重组了一批国有资产。赋予莞韶产业园市级经济管理权限。贯 彻落实中央八项规定精神，开展党的群众路线教育实践活动，促进政风行风好转。建 立健全科学决策、调查研究、督察督办等反“四风”长效机制31项；市政府精简议 事协调机构374个，撤销比例达80. 4%;以市政府名义召开的全市性会议精简50%;

　　以市政府、市政府办公室名义下发的正式文件精简58. 4%;市级考核检查活动精简 90%、评比表彰活动精简52%;市级“三公”经费及会议费减少27. 5%。重点项目 廉政风险防控试点工作有序开展。坚持有腐必反、有贪必肃，严肃查处了一批违法违 纪案件和损害群众利益行为。

　　各位代表，过去一年的成绩来之不易，这是全市人民团结奋斗的结果。我代表市 人民政府，向全市人民，向驻韶部队官兵，向各民主党派、各人民团体和各界人士， 向支持韶关发展的港澳台同胞、海外侨胞和海内外朋友，表示衷心的感谢！

　　同时，我们也要清醒认识存在的问题。主要是：经济增长没有达到预期目标，这 既有经济下行压力加大的客观原因，也有对新常态下经济发展新特征认识不深刻的主 观原因。同时，我市经济发展质量不够高，资源能源约束加大，污染防控形势严峻， 节能减排任务艰巨；中心城区辐射带动能力不强，区域发展不平衡；基本公共服务、 食品药品安全、征地拆迁、社会治安等方面的薄弱环节较多，社会建设任务繁重；创 新创业氛围不够浓厚，政务服务水平与广大群众的要求还有差距，发展环境需要继续 优化。对此，我们将强化责任担当，积极加以解决。

2015年工作安排

　　2015年是我市绿色转型振兴发展的重要一年。世界经济仍处在深度调整期，复苏 进程艰难曲折。国内“三期叠加”压力加大，经济进入中高速增长、结构优化升级、 更加注重创新驱动的新常态。同全国、全省一样，我市经济面临较大下行压力。同时 要看到，世界经济向好，我国经济将继续保持稳定发展态势。省委、省政府对粤东西 北地区振兴发展持续发力，东莞全面对口帮扶强劲给力，我市发展基础不断夯实，广 大干部群众推动发展的正能量不断集聚，保持经济平稳较快增长有基础也有条件。我 们必须坚定信心，防微虑远，趋利避害，牢牢把握发展主动权。

　　今年政府工作的总体要求是：深入贯彻落实党的十八大、十八届三中、四中全会 和习近平总书记系列重要讲话精神，积极适应新常态，紧紧抓住省委、省政府促进粤 东西北地区振兴发展的机遇，强力推改革，巧力促增长，加力调结构，着力惠民生， 在发展中致力转变发展方式，更加注重质量效益，更加注重结构调整，更加注重创新 驱动，更加注重开放合作，更加注重统筹协调，全力推动绿色转型振兴发展。主要预 期目标是：生产总值增长10%左右；人均生产总值增长9%左右；固定资产投资增长 16%;社会消费品零售总额增长11%;地方公共财政预算收入增长与经济增长基本同 步；城镇和农村居民人均可支配收入增长8%;城镇登记失业率控制在3%以内；居 民消费价格指数涨幅控制在3%左右；完成省下达的节能减排等任务。

　　围绕上述目标，今年突出抓好五个方面的工作：

　　一、加强经济建设

　　在推动韶关科学发展的全局里，要始终以经济建设为中心，促进“四化”同步， 着力提质增效升级，推动经济平稳较快发展。

　　强化“三大抓手”继续打好交通基础设施大会战。建成大广高速公路。依法开 展土地和房屋征收工作，解决资本金问题，加快武深、汕昆高速公路建设。改革投融 资机制，创造政策条件，争取在引进社会资本建设韶新、雄乐等高速公路上有所突 破。加快建设武广高铁乐昌东站。加快港口规划建设，确保年内动工改造北江航道。 加快芙蓉新区综合客运枢纽、市区东郊客运枢纽和市区四大出口公交站场建设。完成 国、省道干线改造300公里以上，加快推进国道323线和省道258线亚麻湖至英德段 改造建设。推进韶关机场扩建。做好韶贺柳铁路前期工作。加快旅游公路改造。

　　加快产业园区扩能增效。明确产业定位，促进产业园区化、园区特色化、配套一 体化，构建产业生态圈。加大基础设施和就业、融资、技术、物流等服务平台建设力 度。加快国道323线莞韶产业园段、莞韶大道等道路和园区污水处理厂建设。抓住东 莞对口帮扶韶关的机遇，强化产业项目对接和建设进度，加快粤商高科创新园、权众 集团和东盈服装等重点项目建设，依托智慧螺丝城打造紧固件产业基地。对入园企业 进行调查梳理，建立土地、税收等投入产出台账，按分类处理原则，重点扶持发展潜 力大的优质企业；对未履行土地出让合同或投资合同的企业，采取措施督促整改，倒 逼企业达产提效。拓展园区发展空间，开工建设莞韶城三期，谋划好主城区北部产业 园。争当全省对口帮扶排头兵。积极支持七县（市）与东莞七镇产业对接工作，做优 做强各县（市）产业园区。争取全国老工业基地搬迁改造试点政策，支持韶冶、众力 发电等企业搬迁改造。

　　深入推进城区扩容提质。更加注重规划引领作用，落实“三规”合一。全面推进 芙蓉新区起步区建设，通过交通设施和医院、学校、酒店、公园、专业市场等服务设 施的完善，加快打造产业和人口聚集新平台。加快农民安置房建设进度。强力破解征 拆难题，加快芙蓉隧道、南华路、曲江大道、滨江路和新白线等交通设施建设，通过 绿化、路灯、公共开敞空间建设，打造各具特色和文化内涵的道路。加快市民文化活 动中心等公建项目建设，引进社会资本建设以三甲医院为核心的医疗机构集群。加快 五星级酒店、商业综合体、综合商贸市场、商业街、金融街、总部经济区建设。优先 建设垃圾中转站等公共设施。在老城区，要突出优化城市功能、改善城市形象和治理 “城市病”。新建东环线二期、上饶路、移山路、风华路等，完成芙蓉北路改扩建，同 时探索划设公交车专用道，综合施策缓解交通拥堵问题。加快推进南水水库供水项 目。加快供水、供气、截污管网建设。继续实施一批便民惠民的“五小”工程。加强 背街小巷和桥底环境整治。优化教育、卫生、文化等优质公共资源配置，引导小岛片 区等过度密集地区的机构与人口向外疏散。加快推进西河体育中心地下空间开发建 设，开展地下空间商业开发试点工作。加快城中村改造，推进市区农民新建住房从分 散建设向集中安置转变。鼓励历史街区物业业主按街区保护规划自行开展“三旧改 造”。切实做好新农村建设总体规划，规范农用地使用管理。积极争取广东省新型城 镇化试点。各县（市）要抓好城市新区建设，或者集中连片改造老城区，增强县城和 中心镇的承载能力。

　　着力产业转型升级。编制“十三五”规划和重点专项规划。促进传统优势产业规 模化集聚化发展，不断向中高端迈进，并积极培育新增长点。大力发展现代农业。加 快农业从“低端数量型”向“高端质量型”转变，从“一产独进”向“三产融合” 转型。稳定粮食和主要农产品生产，划定永久性基本农田保护红线。加强农业基础设 施、高标准基本农田、小农水重点县和“五小”水利工程建设。加强农民专业合作组 织建设，大力发展家庭农场。推进土地经营权确权登记颁证试点工作。鼓励农民以互 换方式解决承包地细碎化问题，有序开展农地流转，推进适度规模经营，打造规模化 生产基地。利用主城区农用地发展现代都市休闲体验农业。加强新型职业农民培训。 加快新雪域和市农资专业市场等项目建设。大力发展农村电商，推进农产品零售终端 网络化。支持农产品生产商在城乡设立专卖店或专售区，加强与东莞产销合作，完善 农产品市场体系。大力培育发展林业经济，突出扶持石斛、茶叶、油茶、中药材四大

　　产业，催生扶强一批林下经济龙头企业。

　　力促工业提速增效。围绕产业定位，培育产业链，促进产业聚集。依托机械制造 基础，发展机械装备制造业，特别是在矿山机械、建筑机械等相对优势领域着力打造 几条完整的产业链，发展整机整车生产。依托种烟、烤烟、烟草薄片、卷烟生产、烟 草物流等产业环节比较完整的优势，发展烟草业。以韶钢、韶冶为依托，优化钢铁和 有色金属冶炼产业，重点推进华南特钢循环经济产业基地、有色金属新材料产业基 地。以韶能水电和生物质发电项目、韶关电厂、国电煤矸石电厂、始兴羊角山风力发 电、新丰金竹风力发电、乳源大布大桥风力发电和南雄华电热电联产等项目为依托， 发展电力产业。依托农产品和水资源优势，发展食品饮料业，加快推进娃哈哈、王老 吉凉茶、青岛啤酒和广业甜味剂等项目建设，重点推进广东（曲江）食品产业园区建 设。以丹霞制药、利民制药、东阳光制药等骨干企业为依托，发展制药产业。以旭 日、德宝等企业为基础，发展玩具产业。以鸿伟木业、汉鸿木业、五联木业等企业为 依托，适度发展木材深加工和家具制造业。以南雄和翁源等精细化工产业园为载体， 发展环保涂料产业。以7000吨中色南方稀土（新丰）分离项目建设为依托，规划建 设稀土特色产业园。支持南雄彤置富水泥和曲江台泥、新丰鸿丰水泥扩能增效项目建 设。编制工业新增长点目录，实行一企一策，助力重点龙头企业，扶持小微企业，促 进个体户转企业。新增规模以上工业企业40户。积极做好中小企业服务，全面清理 涉企收费，减轻企业负担。大力引导中小企业与大型企业协作配套，不断延伸产业 链，加快形成大企业“顶天立地”、小企业“铺天盖地”的局面。同时采取措施扶持 建筑业加快发展，拓展市外业务。

　　推动服务业大发展。坚持规划引导与项目带动，促进旅游与文化创意、健康养 生、体育健身等产业融合发展，延长产业链，加快旅游业从观光型向休闲度假型转 型，从追求游客总量向提高游客留宿量转变。按照分区集群成气候的思路，加快构建 龙头旅游基地加配套项目的项目群，打造综合旅游接待平台，增加留宿游客。加快环 丹霞山旅游产业园、南岭国家森林公园生态旅游、云髻山古镇以及五星级酒店等重大 项目建设。大力发展健身步道、自行车绿道、露营地、北江水上观光。组建“县农家 乐指导团”，指导农家乐旅游标准化建设，着力培育发展特色旅游村镇、旅游休闲基 地、户外运动基地。总结南雄银杏观赏旅游营销经验，创新旅游营销与城市形象推广 方式，用好互联网等新媒体，线上线下协调营销。按照“城里建商场、城郊建市场” 的思路，加快市区和县城商业平台建设，培育乡镇商贸中心，发展社区商业；支持鑫 金汇等项目加快建设，打造东环商贸城，推动全市商贸批发市场专业化、规模化、集 聚化、规范化发展。推进成品油保障和物流基地、粤北国际物流园、韶关一类口岸、 南雄精细化工交易中心、新丰横江商贸物流城、中农批电子商务城建设，鼓励大型企 业物流业务本地化。健全城市配送管理机制，规划建设快递产业园，启动快递下乡工 作，促进快递物流业加速发展。以推进芙蓉新区医疗机构集群等项目为抓手，发展健 康医疗服务产业。引导房地产市场和物业管理健康发展。积极发展信息服务业，扶持 韶关（鹏洲）电子商务孵化港、U购网、韶乐购等电商企业做大做强。支持韶关学院 和鑫金汇合作建设电子商务研发中心。依托黄沙坪电商产业园和众瀛大数据公众信息 服务平台，大力发展电子商务，催生新的经济增长点。加快异地灾备数据中心建设， 抓好云计算电子政务公共平台和应用试点示范项目，加快大数据技术应用，逐步解决 部门单位信息互不联通的信息孤岛问题。促进信息消费，加快4G服务广覆盖。建设 完善信用体系，鼓励金融业创新发展，引导扩大信贷投放，大力发展多层次资本市 场，支持具备条件的企业加快上市。全面落实粤东西北地区农村金融发展八项措施， 推进农村金融村村通。

　　扎实推进质量招商和招才引智。继续实施韶关市创新人才引进培养工程，推进十 三个人才子计划的落实。制定产业发展规划，注重预招商规划，建立招商信息库、项 目库、专家库和客商库。制定招商引资行动方案，着力在发挥自身优势、强化产业配 套、创新招商方式、实施精准招商上下功夫，扩大引资成效。大力实施补链招商、强 链招商和扩链招商，动态锁定一批重点目标企业和项目，突出引进行业龙头企业和配 套企业。大力推行以商招商、行业协会招商、莞韶联合招商和中介招商，强化项目跟 踪服务，提高项目履约率和开工率。重视发挥韶关商会作用，促进“金凤回巢”、 “乡贤回归”。完善重大招商项目“绿色通道”工作机制，优化投资软环境，营造 “亲商、扶商、护商、安商”氛围。参加世博米兰展，推介韶关。用好与旧金山、荣 州等地的友城关系，促进韶关发展。

　　深入实施创新驱动战略。引导扶持市民创新创业。推进科研项目与产业需求对 接，力争在机械装备制造、电子信息等关键技术领域，形成一批科技成果。建立工业技改项目库，扩大技改投资，力促3年内主导产业技改面达50%，提高质量效益。用 好韶关学院创新资源，加快发展新型研发机构、高新技术企业、科技型企业。支持组 建一批产业技术创新联盟。

　　二、加强文化建设

　　实施“文化名城”战略，健全公共文化服务体系和文化市场体系，满足市民文化需求。

　　大力培育和践行社会主义核心价值观。广泛宣传“中国梦”和社会主义核心价值 观，加强公民道德和未成年人思想道德建设，推进诚信建设和志愿服务制度化，加大 “扫黄打非”专项整治力度，深化全国文明城市创建。特别要充分激发广大干部群众 不甘落后、创新创业的激情，营造加快发展的良好社会文化氛围。

　　加快特色文化建设。深入挖掘历史人文、生态山水等地方特色文化资源，弘扬、 传承客家文化，推进城市建设与文化建设融合发展，提高城市品位。在城市规划建设 中充分植入韶关特色文化元素，加强特色文化景观和重要区域、重要地段文化标识设 置，突显城市文化主题。围绕以丹霞山为代表的丹霞文化，加快环丹霞山产业园项目 建设；围绕以南华禅寺为代表的禅宗文化，加快大南华文化项目建设；围绕以张九龄 为代表的名人文化，加快文化名人项目建设，加快李汉魂公馆、余汉谋公馆维修改 造，支持涂志伟美术馆的发展；围绕以珠玑古巷为代表的寻根文化，加快珠玑古巷项 目建设；围绕乳源瑶族少数民族文化，支持少数民族继承发展传统体育、舞蹈、歌唱 文化；依托原中央苏区、革命老区，弘扬红色文化。

　　加大文化惠民力度。实施重点文化惠民工程，加快县城数字影院全覆盖，完成农 村广播电视无线覆盖工程建设等任务。大力开展“中国梦•我的梦”、乳源瑶族“十 月朝”、禅宗六祖文化节、青岛啤酒节、百年东街文化节、南雄姓氏文化旅游节、民 间艺术花会等品牌文化活动。加快文化设施达标建设，重点推进芙蓉新城主题文化公 园建设，以及西河文体中心和韶关剧院改造等项目。创新公共文化服务方式，推行 “群众报单、政府买单”的公共文化服务供给方式，破解公共文化供给与需求脱节问 题。深化文化事业单位改革，完善文艺精品创作扶持机制，扶持市直媒体、新兴媒 体、文艺团体和专业剧团创新发展。鼓励民间资本参与公共文化建设，促进公共文化 服务多元化、社会化。

　　加强文化遗产保护和利用。抓好武江南路历史建筑修复工作，修复广富新街、升 平路等历史文化街区，打造东堤艺术字画一条街、百年东街餐饮娱乐购物一站式服务 商业街。推进曹角湾村、下胡古村、湾头村、芙蓉山寺和正觉寺改造。加强对粤北采 茶戏、瑶族刺绣等非物质文化遗产的保护传承。

　　加快文化产业发展。制定全市文化产业项目库，完善文化产业发展规划，着力培 育骨干文化企业，扶持小微文化企业加快发展。大力发展以旭日为龙头的玩具文化产 业。推进科艺创意印刷、南雄罗曼水晶钢琴生产基地、珠玑文化产业园、翁源兰花文 化创意产业园、始兴盛怡制笔产业园、始兴丝绸创意文化产业园、新丰云髻山文化产 业园、乳源民族文化产业园、金鸡岭文化产业园、大南华文化创意产业园建设。加快 培育文化消费市场，提高居民文化消费水平。

　　三、加强社会建设

　　突出富民、惠民、利民、安民导向，不断改善民生福祉，促进社会公平正义，增 强市民幸福感，促进社会和谐。

　　持续增进民生福祉。实施高质量就业计划，加强就业创业扶持，成立青年创业协 会，推进全民创业特别是青年创业示范项目建设。加强社保扩面征缴工作，加大执法 检查力度。做好被征地农民社保实名制工作，推进城乡养老保险制度衔接，尽快完善 城乡医保一体化平台。新开工建设保障房2 7万套，基本建成1.4万套。完善安置房 分配及社区管理制度。培育养老服务多元主体，探索医养融合模式，完善养老服务体 系。提高低保、五保和优抚对象等补助标准，统筹社会救助体系建设，发展社会福利 和慈善事业，建成市社会福利院老年人综合服务大楼，加强恤孤、助残工作。做好原 中央苏区县和革命老区县申报工作。深化扶贫开发“双到”工作，整合涉农资金集中 用于扶贫开发和新村建设，完成3698户农村低收入住房困难户危房改造。稳步推进 城乡客运一体化。加快村村通自来水工程建设。

　　加快发展社会事业。力争实现全市省教育强镇全覆盖。以加强信息化为重点，继 续推进县域内城乡义务教育均衡发展。支持韶关学院内涵发展。加强教育资源特别是 职业技术教育资源整合，支持乳源瑶族自治县创建全国职业教育示范县。优化发展普 通高中教育和特殊教育。推进华师附属韶关中学、韶关实验中学（二期）等项目建 设。认真实施第二期学前教育三年行动计划，提升学前教育整体发展水平。大力发展 民办教育。深化医药卫生体制改革，巩固完善基本药物制度和基层运行新机制。强化 卫生应急、疾病预防控制、妇幼保健和卫生监督工作。积极推动社会办医。夯实人口 计生基础工作。加快粤北医院全科医生培训基地、芙蓉新区三甲医院等项目建设。加 强基层医疗卫生机构标准化建设。引导名医到社区医院轮流坐诊。深入开展全民健身 活动，建成市区四个游泳场以及1个乡镇农民体育健身广场。力争在省第十四届运 动会上取得好成绩。加大农产品源头安全监测体系建设力度，加强食品药品安全监 管，探索建立食品药品黑名单制度。完善安全生产监管体系，提高安全生产水平。加 快气象现代化建设，完善预警信息发布体系。

　　推进社会治理创新。深入推进社会信用体系和市场监管体系建设。推进社区治理 改革。探索成立居民准物业自治管理组织。推进社会组织孵化基地建设，深化政府购 买社工服务试点。推进户籍制度改革，提升流动人口动态服务管理效能。加强劳动者 权益保障，构建和谐劳动关系。加强法治宣传教育，推进法治社会建设。健全法律服 务体系，全面实施政府法律顾问和一村（社区）一法律顾问制度，深入开展法治城 市、法治县（市、区）、法治乡镇（街道）和民主法治示范村（社区）创建活动。大 力推进基层治理法治化，加强行业性、专业性人民调解组织建设。依法强化网络管 理。加强应急指挥平台建设，提高公共安全和防灾减灾能力。完善重大决策社会稳定 风险评估机制，推进社会治安视频监控系统建设，加强信访维稳，深入创建“平安韶 关”。加强“违法建筑、违法用地”查处长效机制建设，规范城乡建设秩序。加强档 案管理和保密工作。争创第十届全国、全省双拥模范城。

　　今年，市政府将在继续抓好以往承诺民生实事的同时，办好九件实事：1.强化 就业培训服务；2提高底线民生保障水平；3.开展山区中小河流整治；4.完成300 公里以上新农村公路路面硬化；5.将城乡居民基本医疗保险补助标准从年人均320元 提高到360兀；6.完成市广播电视台广播电视米编制播高标清升级改造；7.建成市 区小岛片区截污管网；8.建成市区田螺冲污水处理厂一期工程；9.建设芙蓉新城盆 景山公园、武江区移山路公园和黄沙坪公园一期以及浈江区田螺冲棚改安置区矿山 公园。

　　四、加强生态文明建设

　　大力实施主体功能区规划，积极推进国家生态文明先行示范区建设，努力将生态优势转化为经济优势，推进经济社会发展。

　　加强造林绿化工作。加强交通主干道及重要景区、主要流域、城镇周边山体等 “三边”造林工程和森林公园建设，扩大省级生态公益林面积，提升生态景观林带、 碳汇造林、乡村绿化美化工程质量，完成省下达的目标任务。实行护林员薪酬与工作 绩效挂钩。关停整合一批现有木材加工企业。加快苗木基地、森林防火直升机基地建 设。发挥主城区和芙蓉新区的示范带动作用，加强城市绿化美化。借鉴外地经验，鼓 励社会资本参与造林绿化和森林公园建设。

　　提升环境保护水平。实施新环保法，加大环保执法和惩治违法行为力度。建立道 路遗洒、施工扬尘、环境损害、破坏森林、非法采矿等违法行为有奖举报制度，并探 索建立环保警察制度。对损害环境的责任单位和责任人，受到处罚而拒不改正的，按 照原处罚数额按日连续处罚，构成犯罪的依法追究刑事责任。加快推进国家节能减排 财政政策综合示范城市建设，狠抓落后产能退出、供电与产出挂钩和大气、水体污染 减排。加大对PM2.5、PMi的监控和大气污染防治，加强重污染天气监测预警；推行 清洁生产，突出高污染燃料工业小锅炉和工业企业大气污染治理；加强机动车污染防 治，扩大市区黄标车禁行区，加大黄标车和老旧车淘汰力度；推进市区公交电动化项 目，推广应用新能源汽车。加强城市扬尘污染控制，扩大城市禁燃区。推进大宝山、 韶钢环保改造和韶冶整体搬迁升级改造项目。加强水污染防治，加快市区截污管网、 污水处理厂建设及翁源李屋拦泥库外排水处理扩建工程建设。加强生态修复治理，开 展矿山环境综合治理、水土流失治理、石漠化治理和重金属污染防治及受污染土壤治 理修复。加快曲江沙溪民采遗留矿山综合治理，关停淘汰污染重、效益差、产业关联 度低的涉重企业。加强危险废物处置设施建设。以生活垃圾、生活污水、禽畜污染整 治为突破口，大力综合整治农村人居环境。健全突发环境事件应急评估、备案、预警 及处置、环境污染事故预防和应急处理制度，防止重大环境污染发生。

　　提高资源节约集约利用水平。推进循环经济园区、企业建设试点，大力发展循环 经济，提高资源利用效率。落实国家阶梯用水、用电、用气政策。深入落实最严格的 水资源管理制度。严把环保准入关口，对重大项目环评和规划环评提前介入。开展能 源消耗容量分析，合理调节不同能耗的投资项目。实施投入产出约定机制，促进企业 提高投入产出率。发展绿色节能建筑。加强土地供应和供后动态监管，规范国有划拨 土地的收储管理。探索建立绩效用地综合评价体系，对照投资合同、供地合同，对企 业用地情况进行全面排查，以提高土地使用税等经济杠杆倒逼企业提高土地利用效 率。强化“以亩产论英雄”的理念，推进节地换产，加大对闲置土地的处置力度，重 点加大对产业园闲置土地的处置力度，依法解决企业用地多占少用、占而不用的问 题，把闲置、半闲置和低效用地向优质项目转移配置。推进不动产登记和土地利用总 体规划中期修规工作。

　　五、加强政府建设

　　按照推进国家治理体系和治理能力现代化的要求，以深化改革为动力，更好发挥 政府作用，努力建设人民满意政府。

　　建设法治政府。自觉接受人大和政协的监督，办好人大议案、代表建议和政协提 案。认真听取各民主党派、工商联、无党派人士和各人民团体意见。落实市政府工作 规则，健全决策机制。清理行政职权，编制权责清单，规范权力运行。推进相对集中 行使执法权，减少执法层级，推进监管执法职能与技术检验检测职能相对分离，规范 执法行为。深化政府信息公开，完善网络问政，畅通政府与市民信息互通渠道。推进 机构编制实名制管理“全覆盖”。清理超编制、超规格配备干部和“吃空饷”现象。 依法有序解决征地拆迁、企业改制、土地出让等方面历史遗留问题。努力争取地方立 法权。

　　建设效能政府。向社会公布行政审批办事指南和业务手册，推进行政审批标准化 建设。各部门审批权相对集中，并进入行政服务办事大厅。提高网上办事大厅办事深 度。推行企业登记附加承诺的一表并联审批，力争三天办结营业执照、机构代码证、 国税登记证、地税登记证和刻制公章等“四证一章”事项。再造审批流程，探索实行 简单事项立等审批，联办事项一口办理，关联审批一章多效，网上审批一次领证，踏 勘验收统一勘验的审批流程。改进政府督办工作，对重点工作定期回头看，健全抓落 实工作机制。探索差异化绩效考核评价体系，充分调动干事创业积极性。大力整治为 官不为和“庸懒散拖”的行为。

　　建设创新政府。深化乡镇街道行政体制改革，理顺乡镇街道职责关系，完善基层 治理体系。推进公益三类事业单位和经营服务类事业单位转为企业或社会组织。开展 事业单位法人治理结构试点。总结推广市县镇村四级政务服务的“乳源模式”。盘活市属单位的存量资金、资源、资产。完善国有企业风险管控制度。推进财政体制改革创新，建立健全政府预算体系，探索跨年度预算平衡机制。创新财政沉淀资金管理， 提高财政资金使用效益。改革财政资金绩效评审办法，着重考核年初所定的量化绩效 目标的实现程度。建立城市建设基金，积极利用政府与社会资本合作方式（PPP)推 进城市建设。加强政府债务分析，建立债务预警机制，促进城投等平台公司转型发 展。开展企业税务审计，加强税源管理。创新保障性住房建设供应办法，探索“共有 产权”模式和货币安置，帮助住房困难户早圆安居梦。创新社会管理方式，探索大数 据技术在城市管理、财政管理和公共服务等领域的科学运用，开展数字城管试点工 作。积极推进公车改革，稳步推进机关事业单位养老保险制度改革。

　　建设廉洁政府。严格落实中央“八项规定”精神，坚决整治“四风”。坚持有腐 必反、有贪必肃，以“零容忍”态度坚决查处违法违纪行为。围绕政府工作中的廉政 风险点和腐败易发多发领域，加强源头防控。推进政府性投资项目中介机构与行政主 管部门脱钩。推进县镇村“三资”平台建设。建设工程招标、政府采购实行电子招标 投标，杜绝量身定制式招标，截断围标串标的信息源，减少评标专家的自由裁量权， 解决围标串标和价格虚高痼疾。针对建设工程招投标、财政资金管理、土地出让、重 大工程项目建设等重点领域和关键环节，加强事前事中事后全过程监督。强化对政府 部门、事业单位和市属国有资产的审计监督。深化县镇政府“一把手”经济责任 “同步审计”。开展县镇政府交叉审计试点。严禁骗取、套取财政专项资金，严禁私设 小金库或账外账、资产不入帐，严禁以权谋私、索贿受贿。

　　各位代表，让我们在省委、省政府和市委的正确领导下，坚定信心、群策群力、 奋勇争先，为建设幸福美好韶关而努力奋斗！
